# Pathogen trends and paradigm shifts of respiratory infections in children: a 5-year retrospective study from Perugia

**DOI:** 10.1186/s13052-025-02183-5

**Published:** 2026-01-17

**Authors:** Fiumicelli Elena, Nicolì Sofia, Pazzelli Paola, Penta Laura, Biccardi Angela, Camilloni Barbara, Mencacci Antonella, Di Cara Giuseppe, Verrotti Alberto, Valitutti Francesco

**Affiliations:** 1 Department of Medicine and Surgery, University of Perugia, Perugia, Italy; 2 Pediatric Unit, AO Perugia, Perugia, Italy; 3 European Biomedical Research Institute of Salerno, Salerno, Italy

**Keywords:** COVID-19, Virology, Pediatrics, Microbiology, Infectious disease, Respiratory infections, Antimicrobial stewardship

## Abstract

**Background:**

Respiratory infections are one of the most common causes of morbidity and mortality among children. After the COVID-19 pandemic, the epidemiology of respiratory tract infections has changed and has been better traced by more frequent and less expensive molecular and antigenic tests. The main aim of this retrospective study was to evaluate the epidemiology of respiratory infections among children hospitalized in Perugia during the epidemic seasons of 2018–2019 and 2023–2024, comparing clinical severity, hospitalization length, diagnostic and therapeutic interventions.

**Methods:**

We retrospectively analysed hospital records of all patients admitted to our clinic from October 2018 to March 2019 and from October 2023 to March 2024.

**Results:**

In the post-COVID-19 era, hospitalizations for respiratory infections increased proportionally to other hospitalization causes, remaining the most common ones. Length of hospital stay was shorter, with no difference based on age, C-Reactive Protein levels or symptoms at admission. A change of respiratory infection epidemiology after the COVID-19 pandemic has been witnessed also in our setting: *Respiratory Syncytial Virus* and *Influenza* played a central role, displaying earlier peaks and severe clinical pictures. Increased use of molecular testing allowed for prompt etiological diagnoses, likely contributing to shorter inpatient stays and fewer antibiotic prescriptions, thus reducing economic burden for each patient and promoting improved antibiotic stewardship.

**Conclusion:**

In our setting, respiratory infections still accounted for the majority of pediatric hospitalizations during the autumn/winter seasons. A comprehensive testing strategy for etiological diagnosis of respiratory infections in hospitalized patients seemed to be cost-effective by reducing hospital stay and antibiotic prescriptions. This study may inform healthcare policy by emphasizing the importance of etiological diagnosis and the economic burden of inappropriate treatments.

**Supplementary information:**

The online version contains supplementary material available at 10.1186/s13052-025-02183-5.

## Introduction

Respiratory infections are one of the most common causes of morbidity and mortality among children, as well as the first cause of hospital admission in young children [1, 2]. Upper respiratory infections account for 87.5% of all respiratory infections [3, 4].

Most viral respiratory infections are caused by *Respiratory Syncytial Virus, Rhinovirus, Metapneumovirus, Influenza virus* and *Coronaviruses* [5]. *Streptococcus pneumoniae* and *Mycoplasma pneumoniae* are the main bacterial culprits for acute respiratory infections [3, 6].

Bacterial superinfections are known to occur during viral diseases, as viral infections can induce immunosuppression and facilitate bacterial entrance; however, it has recently been demonstrated that viral infections can also follow bacterial ones or, more simply, co-infection can happen as pathogen clusters are transmitted through the same droplets and secretions [7]. The detection of viral co-infections has been studied recently, especially after the Coronavirus Disease 2019 (COVID19) pandemic, and it is still debated if this phenomenon has changed over time and how it could have impacted the severity of respiratory infections [8, 9].

During and after the COVID19 pandemic, the epidemiology of respiratory tract infections has changed: preventive measures such as masks, school closures, “stay-at-home” orders abruptly interrupted the circulation of respiratory pathogens [10]; in the following years, single pathogen spread has been better detected due to more frequent and less expensive molecular and antigenic tests [11, 12], depicting a shifting landscape in pediatric infectious diseases [13].

In recent decades, antibiotic resistance has become a worldwide, rapidly increasing, health threat. One of the main driving factors for antibiotic resistance is inappropriate prescription of antibiotics for viral infections. This could be limited through timely and appropriate microbiological testing to tailor an etiology-based therapy [14]. Furthermore, appropriate microbiological diagnosis could positively impact hospitalization duration and related costs [15].

Within this framework, our retrospective study was aimed to characterize the epidemiology of respiratory infections over time in our unit and its changes after the COVID-19-pandemic, investigating the effects of this unique epidemiologic and social condition. Additionally, we assessed the use of microbiological testing over the past 5 years and its impact on inpatient hospital duration and associated costs.

## Methods

The study protocol was approved by the local Institutional Review Board (IRB) at the University of Perugia, Department of Medicine and Surgery, on June 14, 2024 with protocol number 4805/2024. We collected data from hospital reports about patients admitted to the Pediatric Unit of Perugia Hospital from October 1, 2018 to March 31, 2019 (pre-pandemic) and from October 1, 2023 to March 31, 2024 (post-pandemic).

A total of 1031 patients (496 female; age range: 0–18 years) were hospitalized, 359 from the pre-pandemic period, 672 from the post-pandemic one.

For the study purposes and in order to reduce possible selection bias, inclusion criteria were: -any respiratory infection leading to hospitalization, or any respiratory infection as final diagnosis (either main or secondary diagnosis). All patients with no respiratory infection at admission or during the hospital stay were excluded from the analysis, and this applied also to positive swab tests performed to asymptomatic patients as screening/isolation procedures.

Patients with respiratory infections were divided into two groups using COVID-19 as a watershed: Cohort A was formed by patients from the pre-pandemic period, while Cohort B by the ones from the post-pandemic period.

**Fig. 1:**
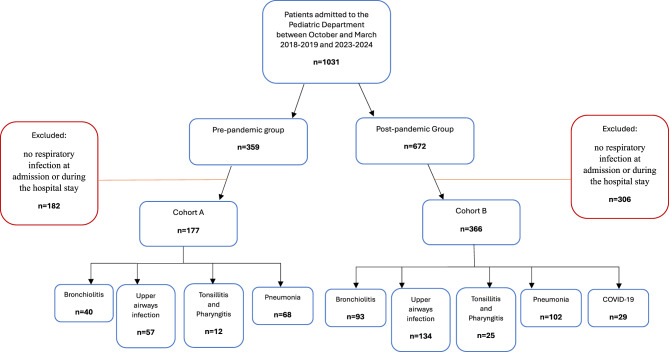
Patient flow diagram of Cohort A and B

A patient flow diagram is summarized in Fig. 1.

We retrospectively analysed these parameters:

- personal and medical history data: age at admission, gender, gestational age, vaccination (including *SARS-CoV-2* vaccination in Cohort B), respiratory diseases, and disability;
- symptoms at admission: fever, respiratory distress, dehydration, and abdominal pain;
- laboratory findings: maximum value of C Reactive Protein (CRP) in mg/dl (positive if > 0.5 mg/dl), microbiological testing (culture, polymerase chain reaction, antigenic testing etc);
- length of hospitalization (expressed in days);
- diagnosis at discharge: diagnoses were categorized into 19 groups, each assigned a numeric code for analysis;
- therapeutic management: oxygen requirement (either low flow or high flow nasal cannula depending on disease and severity) and antibiotics use (intravenous, oral, and whether additional antibiotics were prescribed at discharge);
- cost of hospitalization, estimated through Associazione Ospedali Pediatrici Italiani (AOPI) report regarding standard hospitalization costs [16]. We calculated cost for each patient based on duration of hospitalization and age-adjusted daily cost.

During the pre-pandemic period, microbiological diagnosis relied on viral molecular testing (Seegene AllplexTM Respiratory panel 1- 3, Seegen, Seoul, South Korea) [17], cultural exams and rapid immunochromatographic tests for *Respiratory Syncytial Virus (RSV)*, *Adenovirus* and *Influenza* virus. After COVID-19, all patients admitted with respiratory symptoms or fever underwent rapid molecular testing for *RSV*, *Influenza* and *SARS-CoV-2* (Cepheid Xpert) [17]. When necessary, testing was expanded to include other respiratory viruses (Seegene AllplexTM Respiratory panel 1- 3), atypical bacteria (Seegene AllplexTM Respiratory panel 4), and cultural exams for bacterial pathogens. All these data were anonymised, stored in password-protected Microsoft Excel document and analyzed through software GraphPad-Prism5 (San Diego, CA, USA) in three phases: univariate analysis, interaction between variables and multivariate analysis.

The Kolmogorov-Smirnov test was used to verify normal distribution: as all the parameters analyzed did not have a normal distribution, the Mann-Whitney test was used. Dichotomic variables were analyzed through the Chi square test or Fisher exact test if necessary. Kaplan-Meier curves were generated for hospitalization length in both Cohorts, and differences were assessed using the log-rank test (available in Supplementary figure 1). Finally, linear multivariate regression was performed. *p* values were considered significant when ≤ 0.05 in two-sided test.

This manuscript was drafted in accordance with the STROBE reporting guidelines, and the STROBE reporting checklist is included in Supplement A. Once published, we will develop strategies in order to involve patients and the public in the practical application of our study findings.

## Results

One hundred and seventy-seven (177) patients from the pre-pandemic period (Cohort A) and 366 from the post-pandemic period (Cohort B) were affected by respiratory infections, without significative differences between the two periods; however, when relating patients with respiratory infections in relation to all patients with infectious disease, the prevalence of respiratory infection increased from 66% in pre-pandemic period to 81.33% in post-pandemic period (*p* < 0.0001).

In addition, respiratory infections were further categorized as pneumonia, bronchiolitis, upper airway infections, whose distribution did not change significatively between patients with respiratory infections of the two Cohorts. Demographics, clinical data and respiratory subcategories of the two cohorts with respiratory infections are summarized in Table 1.

**Table 1:** Demographics, clinical data and respiratory infection subcategories of Cohort A and B

	Cohort A (n = 177)	Cohort B (n = 366)	p value
**Age, years [median (IQR)]**	2 (0.3–4)	2 (0.7–5)	0.128
**N of male subjects (%)**	87 (49.2%)	208 (56.8%)	0.092
**N of patients with fever (%)**	125 (70.6%)	239 (65.3%)	0.216
**N of patients with respiratory distress (%)**	77 (43.5%)	155 (42.4%)	0.799
**N of patients with dehydration (%)**	21 (11.9%)	133 (36.3%)	< 0.0001
**CRP, mg/dl [median (IQR)]**	1.9 (0.5–8.4)	1.7 (0–6.4)	0.318
**Hospitalization, days [median (IQR)]**	7 (5–9)	4 (3–6)	< 0.0001
**Hospitalization cost, Euro [median (IQR)]**	6462 (4156–10320)	4156 (3177–6291)	< 0.0001
**N of patients who received swabs (%)**	139 (78.5%)	351 (95.9%)	< 0.0001
**N of patients with bacterial infection (%)**	31 (17.5%)	108 (29.5%)	0.0027
**N of patients who received antibiotics (%)**	144 (81.4%)	231 (63.1%)	< 0.0001
**N of Bronchiolitis (%)**	40 (22.6%)	93 (25.4%)	0.475
**N of Upper Airways Infections (%)**	57 (32.2%)	134 (36.6%)	0.313
**N of Tonsillitis and Pharyngitis (%)**	12 (6.8%)	25 (6.8%)	0.982
**N of Pneumonia (%)**	68 (38.4%)	102 (27.9%)	0.013

In Cohort B, *SARS-CoV-2* was responsible of 8% of admission for respiratory infection and formed a new category. Microbiological diagnosis was done in 117 patients (66.1%) in Cohort A and in 327 patients (89.3%) in Cohort B (*p* < 0.0001). Swabs for respiratory pathogens were performed in 139 patients (78.5%) in Cohort A versus 351 patients (95.9%) in Cohort B (*p* < 0.0001).

As regards bacterial infections, *Streptococcus pneumoniae* (*p* < 0.0001), *Haemophilus influenzae* (*p* < 0.0001), and *Streptococcus pyogenes* (*p* = 0.0048) increased significantly over time. Bacterial infections are summarized in Table 2.

**Table 2:** Bacterial infections

	Cohort A		Cohort B		p value
	N	%	N	%	
***Mycoplasma pneumoniae***	14	7.9	19	5.2	0.250
***Streptococcus pneumoniae***	0	0	38	10.4	** < 0.0001**
***Streptococcus pyogenes***	3	1.9	28	7.6	** < 0.005**
***Staphylococcus aureus***	3	1.9	8	2.2	> 0.999
***Haemophilus influenzae***	0	0	38	10.4	** < 0.0001**

*RSV* was the most frequently reported virus for airway infections, and the virus that most frequently required respiratory support in both Cohorts, even though its rate decreased in Cohort B. In Cohort B, *Influenza A* and *B* (*p* = 0.019), *Metapneumovirus* (*p* = 0.292) and *Rhinovirus* (*p* = 0.015) increased in frequency, although the hospitalization was shorter than patients with same viruses in Cohort A. *Adenovirus* infections decreased in Cohort B (*p* = 0.048).

**Fig. 2:**
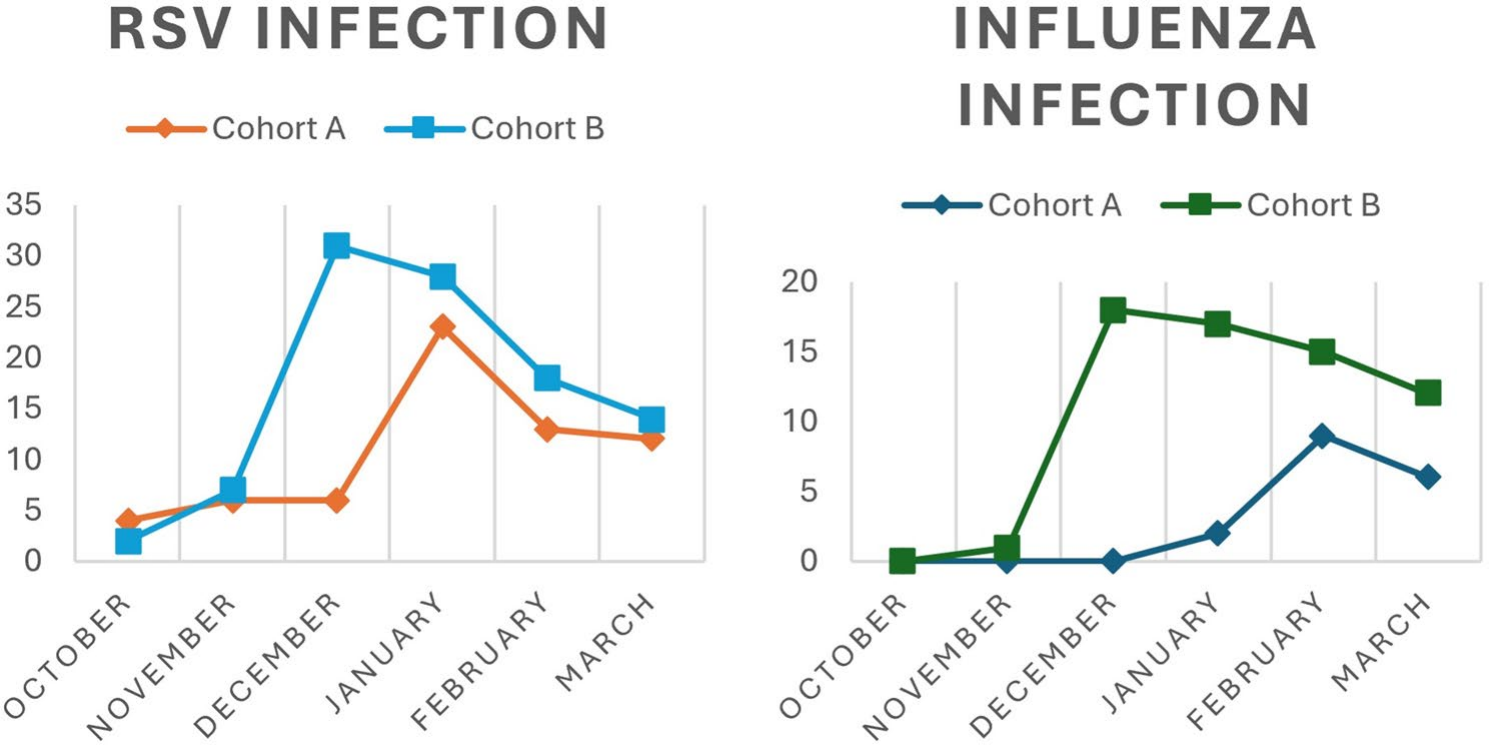
Monthly distribution of RSV and influenza in the two cohorts

The monthly distribution of Influenza virus and RSV was also analyzed between the two periods: both viruses appeared and reached peak levels earlier in Cohort B than in Cohort A, as shown in Fig. 2.

Also, the rate of bacterial infection significantly increased from 17.4% (31 patients) in Cohort A to 29.3% (108 patients) in Cohort B (*p* = 0.0027); on the contrary, antibiotic use decreased: 144 (80.9%) prescriptions in Cohort A and 231 (62.7%) prescriptions in Cohort B were registered (*p* < 0.0001).

The regression analysis showed a positive correlation between antibiotic prescriptions and bacterial infection in cohort B, but not in cohort A.

Among patients with respiratory infections, the median length of hospitalization was shorter in Cohort B (7 days in Cohort A and 4 days in Cohort B, *p* < 0.0001), despite there were no difference in age, symptoms, or CRP at admission. This difference in the length of stay resulted in a median cost reduction of approximately 2306€ (*p* < 0.0001) per patient. These findings were also confirmed when comparing groups of patients with respiratory infection listed as the primary or secondary diagnosis in both Cohorts.

Linear multivariate regression analysis in Cohort A showed that longer inpatient stays correlated with higher CRP value (*p* = 0.0001), viral testing (*p* < 0.0001), detection of bacterial infection (*p* < 0.0001) and antibiotic therapy (*p* < 0.0001).

In Cohort B, male gender (*p* = 0.0006) and CRP values (*p* < 0.0001) correlated with longer hospitalizations in the linear multivariate regression model.

As concerns respiratory support, the rate of patients requiring oxygen was similar between the two cohorts, even when stratifying patients by viral pathogen, as shown in Table 3.

**Table 3:** Oxygen requirements stratified by viral pathogen in both cohorts

VIRUS	Oxygen Requirement Cohort A		Oxygen Requirement Cohort B		p value
	N 57	100%	N 87	100%	
*Rhinovirus*	10	45.5%	22	28.6%	0.195
*RSV*	43	67.2%	58	58%	0.238
*Influenza*	2	11.8%	3	4.8%	0.286
*Parainfluenza*	2	14.3%	5	26.4%	0.670

**Fig. 3:**
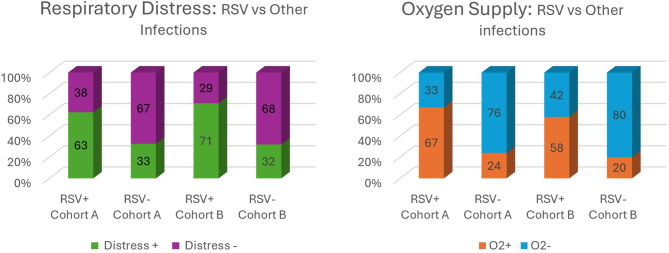
Respiratory distress and need for oxygen supply at the admission

*RSV* confirmed its central role as respiratory pathogen both in Cohort A and B, since it caused more frequently respiratory distress at admission and often required oxygen supplementation, as shown in Fig. 3.

In Cohort B, patients with *RSV* infections had significantly longer duration of hospitalizations than patients with other respiratory infections.

Patients with *RSV* infections in both cohorts had similar ages (*p* = 0.241) and CRP values (*p* = 0.206), but duration of hospitalization was shorter in Cohort B (*p* = 0.0006).

**Fig. 4:**
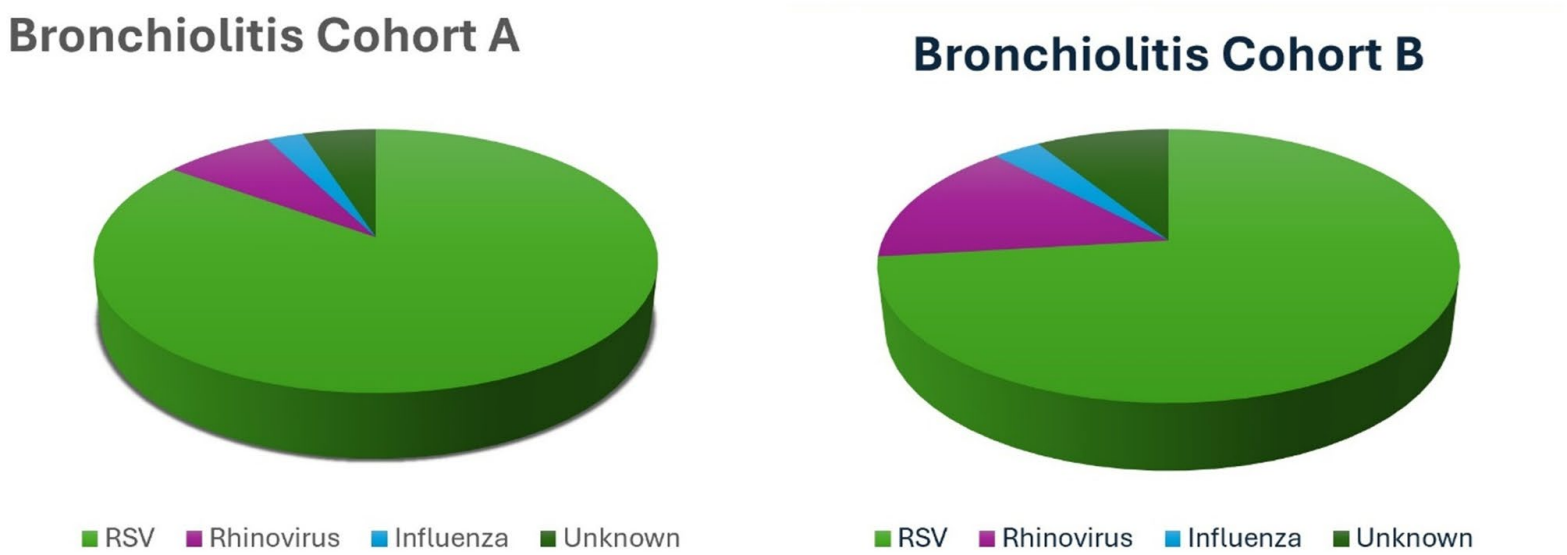
Main causes of bronchiolitis in both cohorts

*RSV* was the main cause of bronchiolitis in both cohorts, and its infection rate remained steady in both cohorts, as for other pathogens (Fig. 4).

Patients diagnosed with bronchiolitis were significantly older in Cohort B than in Cohort A, respectively 0.4 and 0.2 years (*p* = 0.013), and their hospitalization was shorter, 5 days in Cohort B *Vs* 8 days in Cohort A (*p* = 0.0001).

As regards co-infections, patients with multiple infections increased from 22.0% to 34.4% (*p* = 0.0033) from Cohort A to Cohort B. The rate of viral-bacterial co-infections increased from 4.8% in Cohort A to 14.7% in Cohort B (*p* = 0.0003), while the rate of viral-viral co-infections was comparable in the two Cohorts, respectively involving 11% of patients in Cohort A and 13% of patients in Cohort B (*p* = 0.5). In Cohort A, both viral-viral and viral-bacterial co-infections did not show a more severe clinical picture (i.e. oxygen requirement and presence of fever) when compared to single-virus infections. On the other hand, in Cohort B we found a significant higher rate of patients displaying fever when viral-viral coinfection was present (*p* = 0.0129); although oxygen requirement was similar between those having one single virus or more, viral infections (both single-virus or multiple-virus infections) showed higher oxygen requirement when compared to patients with viral-bacterial co-infections (both *p* < 0.05). In Cohort B, rate of antimicrobial therapy was higher in patients with viral-bacterial co-infection compared to single virus infections (*p* < 0.0001) and viral-viral co-infections (*p* < 0.0001).

Hospital length of stay was significantly longer in Cohort A when patients displayed either viral-viral (*p* = 0.0006) or bacterial-viral (*p* = 0.0003) co-infections *Vs* single virus infections, while this difference was not detected in Cohort B. (Available in Supplement B).

## Discussion

In our scenario, regularly addressing etiology of respiratory infections in symptomatic hospitalized patients was valuable as it seemed to reduce hospital stay, costs and antibiotic prescriptions. Respiratory infections were the main cause of hospitalization among our pediatric patients, which is consistent with the findings of current literature [1].

Following the onset of the COVID-19 pandemic, influenza and *RSV* seasonal peaks were not witnessed, due to strict preventive measures such as social isolation and improved sanitization [18, 19].

The significative increase in respiratory infections after the pandemic has been frequently reported, as in our data, and one possible explanation is the “immune debt” [20].

The hypothesis that the population would be immunologically naïve following the COVID-19 pandemic emerged early on, expecting an increase of viral infections among general population and especially in infants born from mothers who have not reinforced their immunity to respiratory viruses, in particular to RSV [21].

Nevertheless, other hypotheses, as increased testing and healthcare-seeking behaviour after daycare/school reopening, should also be considered in explaining the rise of viral infections [22].

Consistent with other reports [23], in our study *RSV* and *Influenza* infections increased, peaked earlier and displayed severe clinical pictures.

These data echo other reports worldwide: “off-season” clusters and longer-lasting infections for *RSV* and *Influenza* were registered [24, 25].

In our experience, both *RSV* and *Influenza* were confirmed as main pathogens both before and after the pandemic.

RSV cases increased in total but, when looking at all the respiratory infections, its ratio slightly decreased; nevertheless, RSV remained the primary pathogen requiring oxygen supplementation and causing respiratory distress. *Influenza*, *Rhinovirus* and *Metapneumovirus* increased both as number of cases and as ratio of the total of respiratory infections.

When focusing on bacterial pathogens, *Haemophilus influenzae, Streptococcus pyogenes,* and *Staphylococcus aureus* were more frequently identified as causes of respiratory infections.

*Streptococcus pyogenes* increase has been similar to the one reported in another Italian study, with M1 subtype as the predominant strain [26].

As regards *Mycoplasma pneumoniae*, it also increased as absolute numbers of cases, but it decreased as rate, contrarily to some other case studies from China where it has recently been responsible for many pneumonia-related hospitalizations [27, 28].

Co-infections were more frequent in Cohort B, especially viral-bacterial co-infections: these were less likely to cause oxygen requirement compared to viral-viral coinfections and single virus infections, albeit exhibiting similar lengths of stay.

In our setting, hospitalizations for respiratory infections increased in parallel with overall hospitalizations: this phenomenon has been also witnessed elsewhere [29, 30]. Nonetheless, Cohorts A and B had similar ages, CRP levels, and symptoms at admission. Patients requiring admission to Pediatric Intensive Care Unit for invasive ventilation were a very minority (3 patients in Cohort A and 5 patients in Cohort B), thus ceasing any statistical analysis and meaningful speculations.

Microbiological diagnoses were obtained for 117 patients (66.1%) in Cohort A and for 327 patients (89.3%) in Cohort B. This significant difference is probably explained by the increased attention to etiological diagnosis, through widespread utilization of microbiological testing (139 tests performed in Cohort A *Vs 351* in Cohort B). In fact, following COVID-19 pandemic, molecular and rapid tests were commonly used at the admission in order to discriminate cases requiring isolation [26, 31].

Particularly, before pandemic pathogen identification was considered useful only in some challenging patients [32, 33], while more recently viral detection swabs were performed in nearly all patients with respiratory symptoms or fever. This trend is somewhat consistent with the correlation between viral search and duration of hospitalization in our Cohort A. According to a recent report, rapid and accurate diagnosis of viral infections could reduce the use of empirical antibiotic usage and improve diagnostic workup/therapeutic decision making [34].

The rate of etiological diagnoses in bacterial infections increased from 17.5% in Cohort A to 29.5% in Cohort B, leading to a reduction of inappropriate antimicrobial therapy. In our opinion, this was a definite step-forward in antibiotic stewardship in our setting, since it has been highlighted elsewhere that one out of four children hospitalized for RSV receives antibiotics [35]. In addition, etiological diagnosis improved the management of patients, as shown by other studies highlighting that the identification of the microbial culprit helped both shortening in-patient stay and avoiding antibiotics use [36–38]. This is also supported by the reduced antibiotic prescriptions in patients displaying one or more viral infection compared to those displaying viral-bacterial coinfection in Cohort B.

Given our results, we hypothesize that clear awareness of infection etiology by molecular testing, especially in cases of confirmed viral infection, could ease physicians to dismiss patients more safely, thus leading to reduced inpatient stay and therefore reduced costs. In fact, in our study hospitalization was shorter in Cohort B (4 days) than in Cohort A (7 days), with a tremendous reduction of the economic burden for each patient, both from the hospital budget perspective and the overall economic impact of parental work leave.

This result is probably also linked to the prompt reports obtained through rapid viral molecular testing performed on every febrile patient. According to other studies, this method often resulted more accurate than antigenic or regular molecular tests [34, 39], albeit another report did not prove this trend [40].

We also acknowledge few limitations of our study: as retrospective single-center design, it is subject to potential confounders such as changes in diagnostic protocols and some medical staff turnover across the study periods.

By including only patients with clinical features of respiratory infection, we minimized detection bias in both cohorts, despite a higher awareness towards respiratory infections by medical staff managing Cohort B could not be excluded.

Our cost analysis has some limitations too: in fact, we estimated costs considering duration of hospitalization and age of the patient, using AOPI report, a pediatric benchmark for Italian Health System. Even though details about procedures, consultations, patient frailties and medications were not considerable for our cost analysis, the AOPI reference could better describe a nationwide trend of health expenditure, equalising possible confounders.

## Conclusion

Our retrospective study revealed a change in the epidemiology of viral respiratory infections, with RSV and influenza infections exhibiting earlier peaks and severe clinical presentations in the post-pandemic period. Respiratory infections remained the major cause of hospitalizations in a pediatric inpatient unit during the autumn/winter seasons, but molecular testing allowed prompt diagnoses, which were likely linked to shorter inpatient stay and fewer antibiotic prescriptions. In our scenario, the cost-effectiveness of massive testing strategy for respiratory infections in hospitalized patients has been highlighted. Whether this also applies to other countries with different health systems or to low-resource settings remains to be addressed in further studies.

## Electronic supplementary material

Below is the link to the electronic supplementary material.

Supplementary Material 1

Supplementary Material 2

Supplementary Material 3. Supplementary figure 1: comparison between Cohort A and B length of hospitalization (Kaplan-Meier Curves)

## Data Availability

The data that support the findings of this study are available from the corresponding author upon reasonable request.

## Abbreviations

AOPI: Associazione Ospedali Pediatrici Italiani
COVID-19: Coronavirus Disease 2019
CRP: C Reactive Protein
IQR: Interquartile Range
O2: Oxygen
RSV: Respiratory Syncytial Virus
SARS-CoV2: Severe Acute Respiratory Syndrome Coronavirus 2
