# Supplementary material for: Pathogen trends and paradigm shifts of respiratory infections in children: a 5-year retrospective study from Perugia
Source: Ital J Pediatr. 2026 Jan 17;52:28. doi: 10.1186/s13052-025-02183-5 (PMC12896027; doi:10.1186/s13052-025-02183-5)
Supplement: Supplementary file 2 — Supplementary Material 2 [file 13052_2025_2183_MOESM2_ESM.docx]

|  | **Cohort A (n=177)** | **Cohort B (n=366)** | **p value** |
| --- | --- | --- | --- |
| **N of patients with Multiple Pathogen Infection (%)** | 39 (22%) | 126 (34.4%) | **0.0033** |
| **N of patients with Single Virus Infection (%)** | 88 (49.7%) | 189 (51.6%) | 0.6745 |
| **N of patients with Viral Co-Infection (%)** | 24 (13.56%) | 43 (11.75%) | 0.5476 |
| **N of patients with Viral-Bacterial Co-Infection (%)** | 8 (4.52%) | 54 (14.75%) | **0.0003** |

**Cohort A and B: co-infections subtypes.**


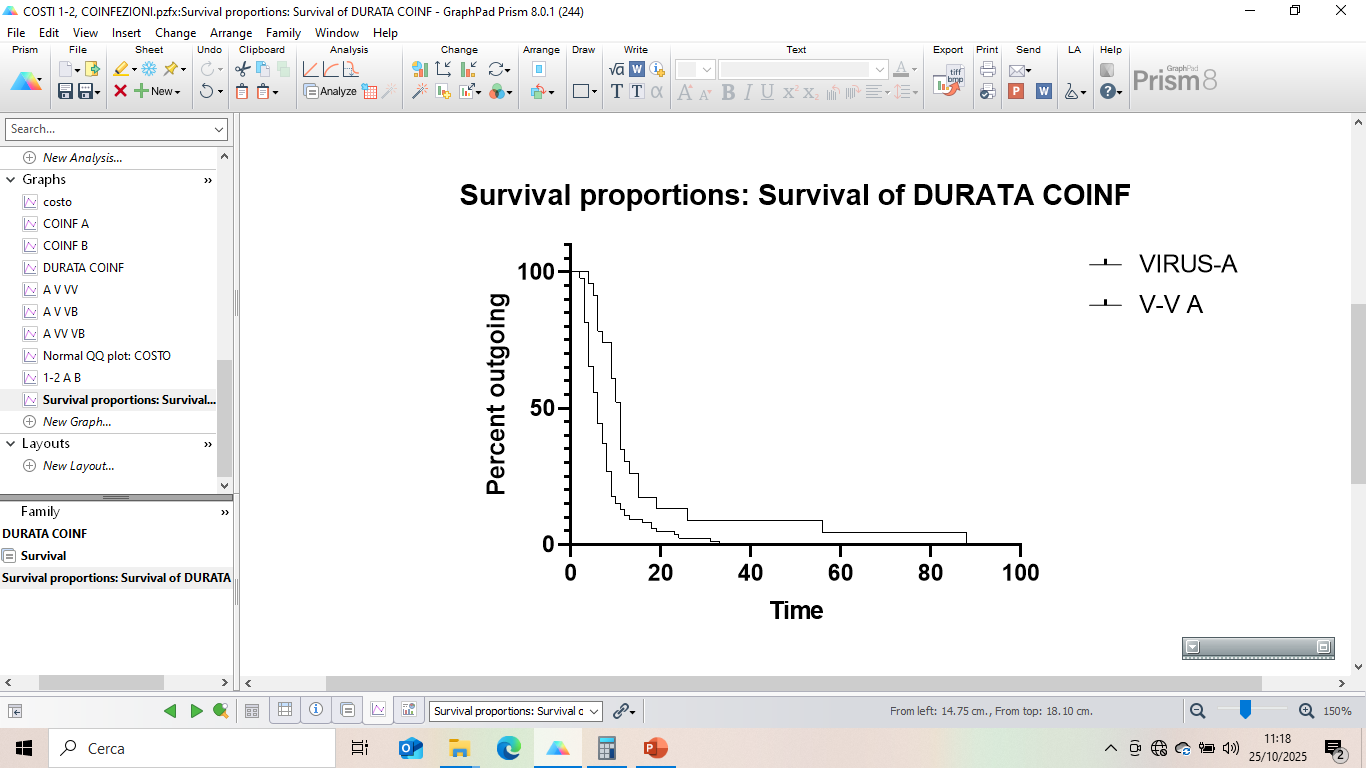
**Cohort A: co-infections and length of hospitalization*.***

Hospitalized patients

Patients displaying viral co-infection had longer hospitalization length than single viral infection. (p=0.0006)

Patients displaying viral bacterial co-infection had longer hospitalization length than single viral infection. (p=0.0330)

Days


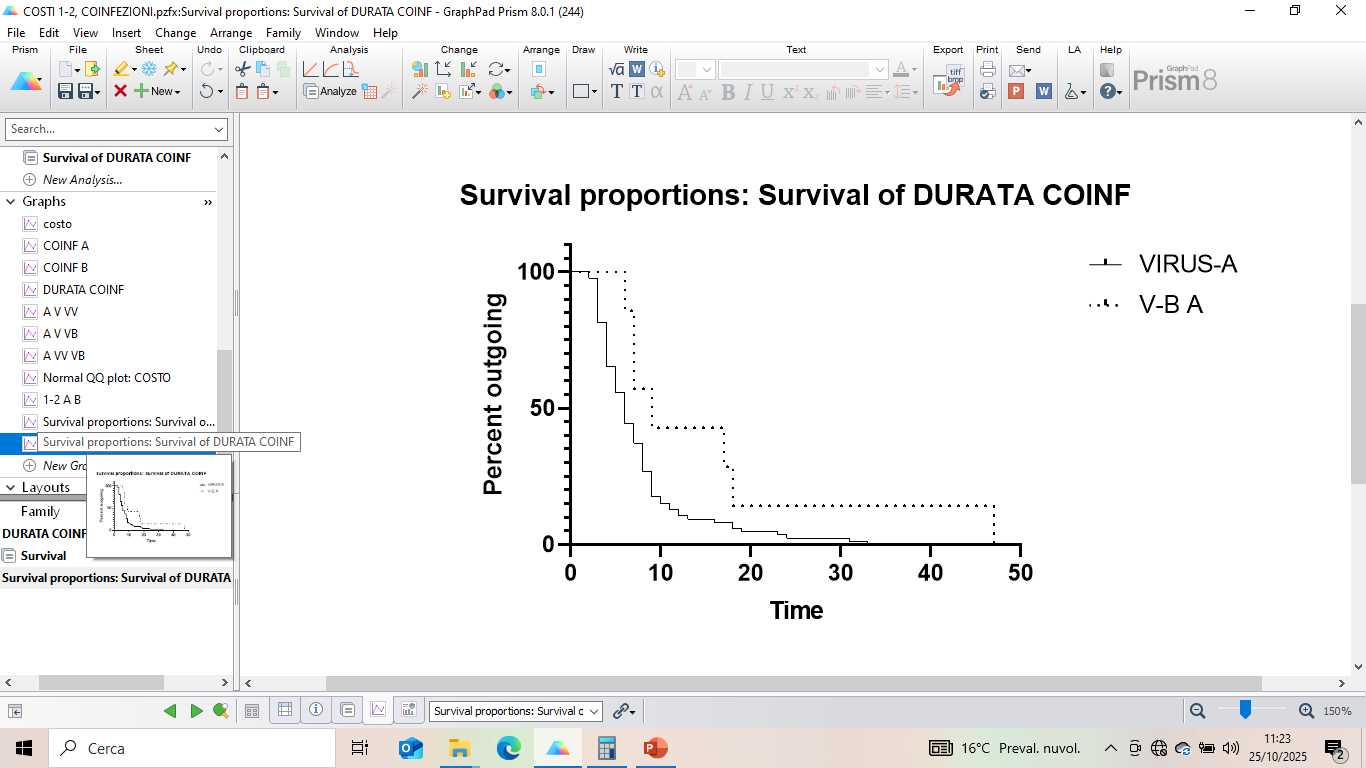


Hospitalized patients

Days

**Cohort A: clinical presentation and treatment required**

|  | **Single virus infection** | **Viral-viral co-infection** | **p value** |
| --- | --- | --- | --- |
| **N of patients with Fever(%)** | 57 (65.5%) | 16 (69.6%) | 0.8074 |
| **N of patients requiring Oxygen (%)** | 40 (46%) | 12 (52.2%) | 0.6439 |
| **N of patients with antimicrobial therapy (%)** | 62 (70.5%) | 22 (95.7%) | **0.0126** |

|  | **Single virus infection** | **Viral-bacterial co-infection** | **p value** |
| --- | --- | --- | --- |
| **N of patients with Fever(%)** | 57 (65.5%) | 6 (85.7%) | 0.4191 |
| **N of patients requiring Oxygen (%)** | 40 (46%) | 4 (57.1%) | 0.7020 |
| **N of patients with antimicrobial therapy (%)** | 62 (70.5%) | 7 (100%) | 0.1840 |

|  | **Viral co-infection** | **Viral-bacterial co-infection** | **p value** |
| --- | --- | --- | --- |
| **N of patients with Fever (%)** | 16 (69.6%) | 6 (85.7%) | 0.6378 |
| **N of patients requiring Oxygen (%)** | 12 (52.2%) | 4 (57.1%) | >0.9999 |
| **N of patients with antimicrobial therapy (%)** | 22 (95.7%) | 7 (100%) | >0.9999 |

**Cohort B: clinical presentation and treatment required**

|  | **Single virus infection (n=188)** | **Viral-viral co-infection (n=42)** | **p value** |
| --- | --- | --- | --- |
| **N of patients with Fever (%)** | 114 (60.6%) | 34 (81%) | **0.0129** |
| **N of patients requiring Oxygen (%)** | 68 (36.2%) | 18 (42.9%) | 0.4181 |
| **N of patients with antimicrobial therapy (%)** | 91 (48.4%) | 25 (61%) | 0.1446 |

|  | **Single virus infection (n=188)** | **Viral-bacterial co-infection (n=53)** | **p value** |
| --- | --- | --- | --- |
| **N of patients with Fever (%)** | 114 (60.6%) | 35 (66%) | 0.4748 |
| **N of patients requiring Oxygen (%)** | 68 (36.2%) | 6 (11.3%) | **0.0004** |
| **N of patients with antimicrobial therapy (%)** | 91 (48.4%) | 51 (96.2%) | **<0.0001** |

|  | **Viral co-infection (n=42)** | **Viral-bacterial co-infection (n=53)** | **p value** |
| --- | --- | --- | --- |
| **N of patients with Fever (%)** | 34 (81%) | 35 (66%) | 0.1638 |
| **N of patients requiring Oxygen (%)** | 18 (42.8%) | 6 (11.3%) | **0.0007** |
| **N of patients with antimicrobial therapy (%)** | 25 (61%) | 51 (96.2%) | **<0.0001** |
