# Supplementary figures and images for: Pathogen trends and paradigm shifts of respiratory infections in children: a 5-year retrospective study from Perugia
Source: Ital J Pediatr. 2026 Jan 17;52:28. doi: 10.1186/s13052-025-02183-5 (PMC12896027; doi:10.1186/s13052-025-02183-5)

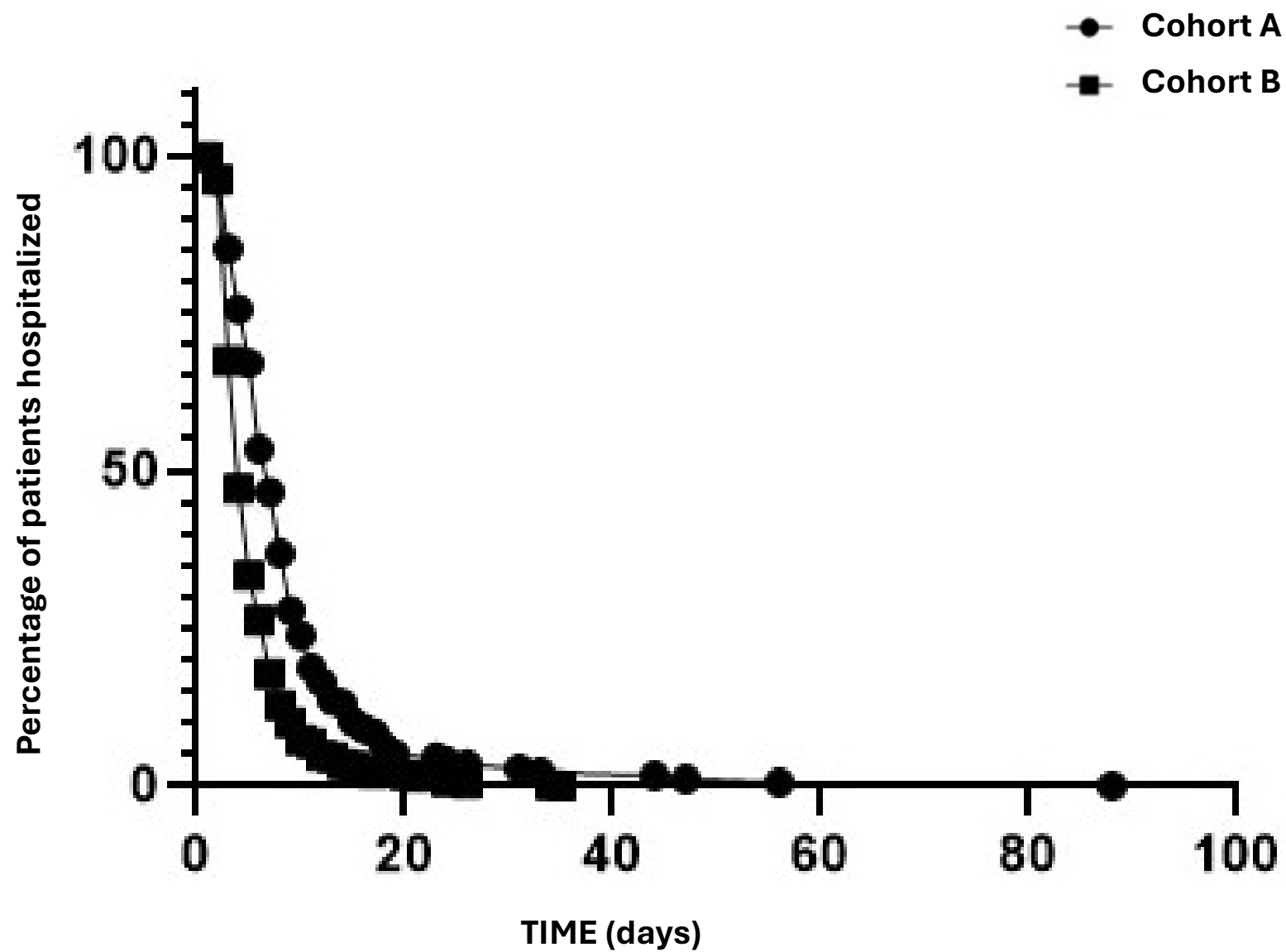

Supplement: Supplementary file 3 — Supplementary Material 3. Supplementary figure 1: comparison between Cohort A and B length of hospitalization (Kaplan-Meier Curves) [file 13052_2025_2183_MOESM3_ESM.pdf]
